# Reproducibility and validity of healthy dietary indices derived from 24-hour dietary recalls

**DOI:** 10.3389/fnut.2026.1730154

**Published:** 2026-04-02

**Authors:** Meiling Li, Guobing Sun, Yu Han, Hong Xu, Junyi Zhang, Tingting Wang, Young Lyu, Hongjuan Cao, Xiude Li, Xinghao Ma, Zhuang Zhang, Wanshui Yang

**Affiliations:** 1 Department of Nutrition, Center for Big Data and Population Health of IHM, School of Public Health, Anhui Medical University, Hefei, China; 2 Department of Physical Examination Center, Lu'an Hospital of Anhui Medical University, Lu'an, China; 3 Department of Chronic Non-Communicable Diseases Prevention and Control, Lu'an Municipal Center for Disease Control and Prevention, Lu'an, China; 4 Department of Clinical Nutrition, The First Affiliated Hospital of Anhui Medical University, Hefei, China; 5 Department of Clinical Nutrition, Lu'an Hospital of Anhui Medical University, Lu'an, China

**Keywords:** 24-hour dietary recall, alternate Mediterranean diet, dietary approaches to stop hypertension, healthy eating index-2015, validation study

## Abstract

**Background:**

Healthy dietary indices are commonly used to investigate diet-disease associations, but the reproducibility and validity of these indices from 24-h dietary recalls (24HDRs) are undetermined. We aimed to investigate optimum number of 24HDRs for measuring healthy dietary indices including the Alternate Mediterranean Diet (AMED), Dietary Approaches to Stop Hypertension (DASH), and Healthy Eating Index-2015 (HEI-2015) in epidemiological studies.

**Methods:**

From July 2021 to July 2022, 432 participants (median age: 48y, 57.2% women) completed 12-day 24HDRs, comprising four quarterly sets of three consecutive 24HDRs (two weekdays and one weekend day in each season). Energy-adjusted intraclass correlation coefficient (ICC) was used to evaluate reproducibility, while energy-adjusted deattenuated Spearman correlation coefficient (rc) were used to examine validity by comparing the estimates against those from the average of 12 24HDRs.

**Results:**

The reproducibility and validity coefficient increased with the increasing number of 24HDRs. We observed a “moderate” reproducibility for three non-consecutive 24HDRs in assessing AMED, DASH and HEI-2015 (ICCs: 0.40-0.43). Healthy dietary indices from one 24HDR showed “moderate” validity (rc: 0.42-0.55). Higher body mass index, female sex, and autumn/winter of administration were associated with low reproducibility of AMED and/or HEI-2015.

**Conclusion:**

Our results support that three non-consecutive 24HDRs provide acceptable estimates of AMED, DASH, and HEI-2015.

## 1 Introduction

In nutritional epidemiology, healthy dietary indices including the Alternate Mediterranean Diet (AMED) score, the Dietary Approaches to Stop Hypertension (DASH) score, and the Healthy Eating Index-2015 (HEI-2015) are commonly used to examine the diet-disease association (1–5). These dietary indices reflect overall dietary quality and the synergistic effects of various nutrients and food components, offering a comprehensive assessment of diet in contrast to focusing on single nutrients (6), and emphasize the consumption of foods like fruits, nuts, vegetables, legumes, whole grains, fish, low-fat dairy, and moderate alcohol intake, while limiting the intake of foods like red and processed meats, refined grains, sugar-sweetened beverages, sodium, added sugars, and saturated fats.

Cohort studies showed that higher adherence to AMED, DASH, and HEI-2015 was associated with lower risk of cardiovascular disease (2, 3), several cancers (4, 5), and overall mortality (7–11). Most of these studies (2–5, 7–9) used food frequency questionnaires (FFQs) to assess long-term dietary intake, while few study has evaluated the reproducibility and validity of FFQs for assessing healthy dietary indices (12). Nowadays, two or more 24-hour dietary recalls (24HDRs) are being increasingly used to measure diet in large-scale epidemiological studies and national nutrition surveys (13). Moreover, several previous studies have used 24HDR-based assessment of healthy dietary indices and investigated their associations with multiple health outcomes (9, 14–17). FFQs rely on participants' ability to recall mean food intake over long intervals (e.g., 12 months) with limited food items, while 24HDRs allow for the collection of detailed information on all foods consumed within the previous 24 h, which do not require a high level of literacy. However, a single 24HDR may not well capture a habitual diet, while multiple 24HDRs place a heavy burden on both participants and investigators. Thus, there is an urgent need to identify the optimum number of 24HDRs to accurately assess long-term adherence to healthy dietary patterns in epidemiological studies. Although several studies (18–24) have assessed the reproducibility and/or validity of nutrients, food groups, dietary patterns (including low-carbohydrate diets and low-fat diets), and meal timing from different number of 24HDRs, to our knowledge, no validation study has evaluated the performance of 24HDR-based measures of AMED, DASH, and HEI-2015.

Herein, we evaluated the reproducibility and validity of AMED, DASH, and HEI-2015 estimated from different number of 24HDRs to determine the optimal number of recalls required in epidemiological studies. We used the average of 12-day dietary recalls (i.e., four quarterly three consecutive 24HDRs, including two weekdays and one weekend day in each season) as the reference method to account for the day-to-day variation in diet (e.g., day of the week and seasonal effect). Additionally, we performed a stratified analysis by age, sex, body mass index (BMI), day of the week, and season of 24HDR administration to investigate factors that might affect the performance of three 24HDRs in measuring healthy dietary indices.

## 2 Materials and methods

### 2.1 Study population

The Anhui Liver Disease Study (ALDS) is an ongoing community-based cohort study started in 2020 to 2022, enrolling nearly 6,200 adults aged 18 years or older in Anhui Province, Central-Eastern China (25, 26). The ALDS aims to investigate diet and other lifestyle behaviors, host genetics, and gut microbiome in relation to the onset and regression of chronic liver diseases. The overall design and sampling framework of the ALDS have been published previously (25). The ALDS employed a multistage cluster random sampling design to enhance population representativeness. Briefly, within Lu'an City, four study sites (Huoshan County, Shucheng County, Jin'an District, and Yu'an District) were randomly selected from all counties and districts, followed by the random selection of four towns or streets within each region and four villages or communities within each town or street. Within each village or community, one residential group was randomly chosen, and 50 households were randomly sampled from each residential group. One adult aged ≥18 years was then randomly selected from each household using the Kish selection grid method. In total, 3,200 individuals were invited, of whom 2,942 completed the baseline survey (participation rate: 91.9%).

From July 2021 to July 2022, the Anhui Lifestyle Validation Study (ALVS) was conducted to validate questionnaires administrated in the ALDS and other cohorts in Anhui. The ALVS is a 1-year longitudinal study that comprised 892 participants including 754 participants from the ALDS and 138 volunteers from other cities in Anhui. Diet was collected at the start and at the end of the study using FFQs, and was also assessed using 12 24HDRs that consist of four quarterly three consecutive 24HDRs (i.e., two weekdays and one weekend day in each season) during the 1-year study period, which has considered the day of the week and seasonal effects in dietary measurement.

In this study, participants were selected from 754 participants in the ALVS (a subset of the ALDS). Participants who did not complete the 12 24HDRs (*n* = 295) or had implausible energy intake (*n* = 27, defined as energy intake above the 97.5th percentile or below the 2.5th percentile) were excluded. Thus, a total of 432 participants were included in the final analysis (Supplementary Figure S1). The study protocol was approved by the Ethics Committee of Anhui Medical University (Protocol Number: 20210730). All participants provided informed consent before enrollment.

### 2.2 Dietary assessment

Detailed instructions for the 24HDR were given to participants prior to the interview. Four quarterly three consecutive 24HDRs were conducted via telephone by trained and experienced interviewers, who were supervised by two registered dieticians, using the USDA Automated Multiple-Pass Method (AMPM) (27). The AMPM was developed to enhance the comprehensiveness and accuracy of data collection, while reducing respondents' burden. Each 24HDR recorded the individual dietary intake for a single day. All drinks and foods consumed in the past 24 h were recalled by participants, with details on the occasions and timing of consumption to capture all eating periods. In addition, participants were required to specify the ingredients and their average proportions for mixed dishes, considering the large difference in household recipes even in the same survey areas. To aid in estimation of food and drink portion sizes, a food-amount booklet was distributed to participants in advance and used during 24HDR interview. In addition, participants completed two face-to-face food frequency questionnaire interviews administered 1 year apart, before and after the 12 repeated 24HDRs, during which standardized food models were used to assist portion-size estimation. This in-person exposure facilitated familiarity with portion-size concepts for subsequent telephone-based 24HDRs.

To account for the seasonal variation in dietary intake, the 1st, 2nd, 3rd, and 4th three consecutive 24HDRs were conducted over four seasons in October 2021, January 2022, April 2022, and June 2022, respectively. Given that diet may vary considerably from weekdays to weekend days, one of two types of three consecutive 24HDRs (i.e., Thursday, Friday, and Saturday vs. Sunday, Monday, and Tuesday) was randomly assigned to each participant during each season. Nutrients were derived using the Standard Edition of the Chinese Food Composition Table.

### 2.3 Assessment of healthy dietary indices

The methods for calculating healthy dietary indices have been described elsewhere (5, 28–30). The AMED score consists of nine components and ranges from 0 to 9 points. The DASH score includes eight components, with a range of 8 to 40 points. The HEI-2015 comprises nine adequacy and three moderation components, ranging from 0 to 90 points. Detailed methods are provided in the Supplementary method 1 and Supplementary Tables S1–S3. Higher scores indicate greater adherence to healthy dietary patterns.

### 2.4 Covariates

A self-reported questionnaire was administered at baseline to collect demographic and lifestyle information, such as age, sex, marital status, education level, alcohol drinking, smoking status, and annual household income. Height and weight were measured using calibrated height-weight scales by trained investigators in compliance with standard operating procedures. BMI was calculated by dividing body weight (kg) by the square of the height (m^2^).

### 2.5 Statistical analysis

We randomly selected six sets of 24HDRs survey including a single 24HDR, 2, 3, 4, 5, and 6 non-consecutive 24HDRs from 12-day recalls over 1 year, to better simulate dietary recall in real-world settings. The average estimates of three healthy dietary indices were then calculated from the six sets of dietary recalls. The AMED and DASH scores were energy-adjusted using the residual method to reduce measurement error (31), whereas HEI-2015 was not energy-adjusted because it is inherently density-based. We used intraclass correlation coefficient (ICC) to evaluate the reproducibility of healthy dietary indices estimated from 2, 3, 4, 5, and 6 non-consecutive 24HDRs (32). Spearman correlation coefficients were used to assess the validity of healthy dietary indices, through comparing the estimates derived from the different number of 24HDRs against those from the average of 12 24HDRs over 1-year. To calculate 95% confidence intervals (CIs) for the reproducibility and validity coefficients, participant-wise bootstrapping with 1,000 iterations was performed. We also calculated *r*_c_ and 95% CIs that were deattenuated for random measurement error in multiple 24HDRs using the formula provided in the Supplementary method 2 (33–35). The level of reproducibility and validity were defined as follows: “weak” (correlation coefficient <0.40), “moderate” (correlation coefficient: 0.40–0.69), “strong” (correlation coefficient: 0.70–0.89), and “very strong” (correlation coefficient ≥0.90) (36). Bland-Altman plot (37) was used to visually assess the agreement and observe trend in overestimation or underestimation of healthy dietary indices in three 24HDRs relative to those in 12 24HDRs. The 95% limit of agreement (LOA) was estimated as the mean difference ± 1.96 standard deviations.

To identify potential factors that may affect the performance of three 24HDRs in assessing healthy dietary indices, we also reported the reproducibility and validity coefficients according to age (<45 or ≥45 years), sex (men or women), BMI (<24 or ≥24 kg/m^2^) (38), day of the week (weekdays or weekends), and season (autumn/winter or spring/summer). The difference was defined as the median difference between estimates based on different numbers of dietary recalls and the reference method (12 24HDRs); Diff (%) was calculated as the absolute difference relative to the median from 12 recalls. To whether these observed subgroup differences exceeded random variation, we further applied bootstrap permutation tests.

All analyses were performed using R (version 4.3.3) and its packages including boot, irr, plm, ggExtra, ggplot2, and cor function, and two-sided *p* < 0.05 were considered statistically significant.

## 3 Results

### 3.1 Characteristics of participants

A total of 432 participants (median age 48 years, IQR: 34–58 y, 247 women) were included in the final analysis. Participants with higher healthy dietary indices were older, were more likely to be married, had lower BMI and higher physical activity levels, and were more likely to be never smokers (Table 1). The median score of healthy dietary indices estimated from different number of dietary recalls and 12 24HDRs (reference method) were similar (difference within 30%, Supplementary Table S4).

**Table 1:** Characteristics of the participants (*n* = 432) by tertiles of healthy dietary indices in the Anhui lifestyle validation study (ALVS, 2021–2022)^a^^b^.

Characteristic	AMED		DASH		HEI-2015	
	T1	T3	T1	T3	T1	T3
Healthy dietary indices, score	2.7 (2.5–4.4)	8.3 (6.6–8.5)	18.0 (15.8–19.9)	30.1 (28.1–33.9)	33.0 (32.0–33.9)	42.8 (41.0–45.0)
Age, years, %					
• 18–39	41.0	25.2	43.1	28.7	33.3	34.3
• 40–59	43.8	46.9	41.7	44.8	43.5	39.8
• ≥60	15.3	28.0	15.3	26.6	23.2	25.9
Women, %	56.3	66.7	59.0	52.8	47.2	61.5
Married, %	78.9	83.2	77.6	86.7	78.3	83.8
Education level, %					
• Primary school or below	37.5	50.0	38.9	37.5	45.4	40.4
• Junior high school	25.7	21.5	24.3	24.3	21.3	17.4
• Senior high school or above	36.8	28.5	36.8	38.2	33.3	42.2
Physical activities, METS-h/week	126 (100–163)	141 (110–176)	129 (103–170)	139 (113–183)	124 (96–163)	130 (103–167)
BMI, kg/m^2^, %					
• <24.0	52.8	50.0	49.3	47.9	45.4	45.9
• 24.0–27.9	29.9	33.3	31.9	37.5	37.0	38.5
• ≥28.0	17.4	16.7	18.8	14.6	17.6	15.6
Income, %^c^					
• Low	31.7	39.9	29.6	33.1	44.8	33.3
• Middle	33.8	27.5	29.6	27.3	22.9	27.5
• High	34.5	32.6	40.7	39.6	32.4	39.2
Total energy intake, kcal/day	1,887 (1,641–2,130)	1,699 (1,491–1,917)	1,763 (1,521–2,132)	1,792 (1,550–2,126)	1,822 (1,654–2,126)	1,780 (1,493–2,091)
Never smoker, %	71.3	78.5	74.8	75.0	66.7	76.9
Never drinker, %	84.9	83.6	84.8	81.3	78.1	85.3

ALVS, Anhui lifestyle validation study; AMED, alternate Mediterranean diet; BMI, body mass index; DASH, dietary approaches to stop hypertension; HEI-2015, healthy eating index-2015.

^a^Tertiles were based on energy-adjusted healthy dietary indices.

^b^Continuous variables are expressed as median (interquartile range), while categorical variables are presented as proportion (%). Values of polytomous variables may not sum to 100% due to missing values or rounding.

^c^Low: 10,000 Yuan per person per year or less; Middle: 10,000–19,999 Yuan per person per year; High: 20,000 Yuan per person per year or more.

### 3.2 The reproducibility of 2–6 non-consecutive 24HDRs

**Figure 1:**
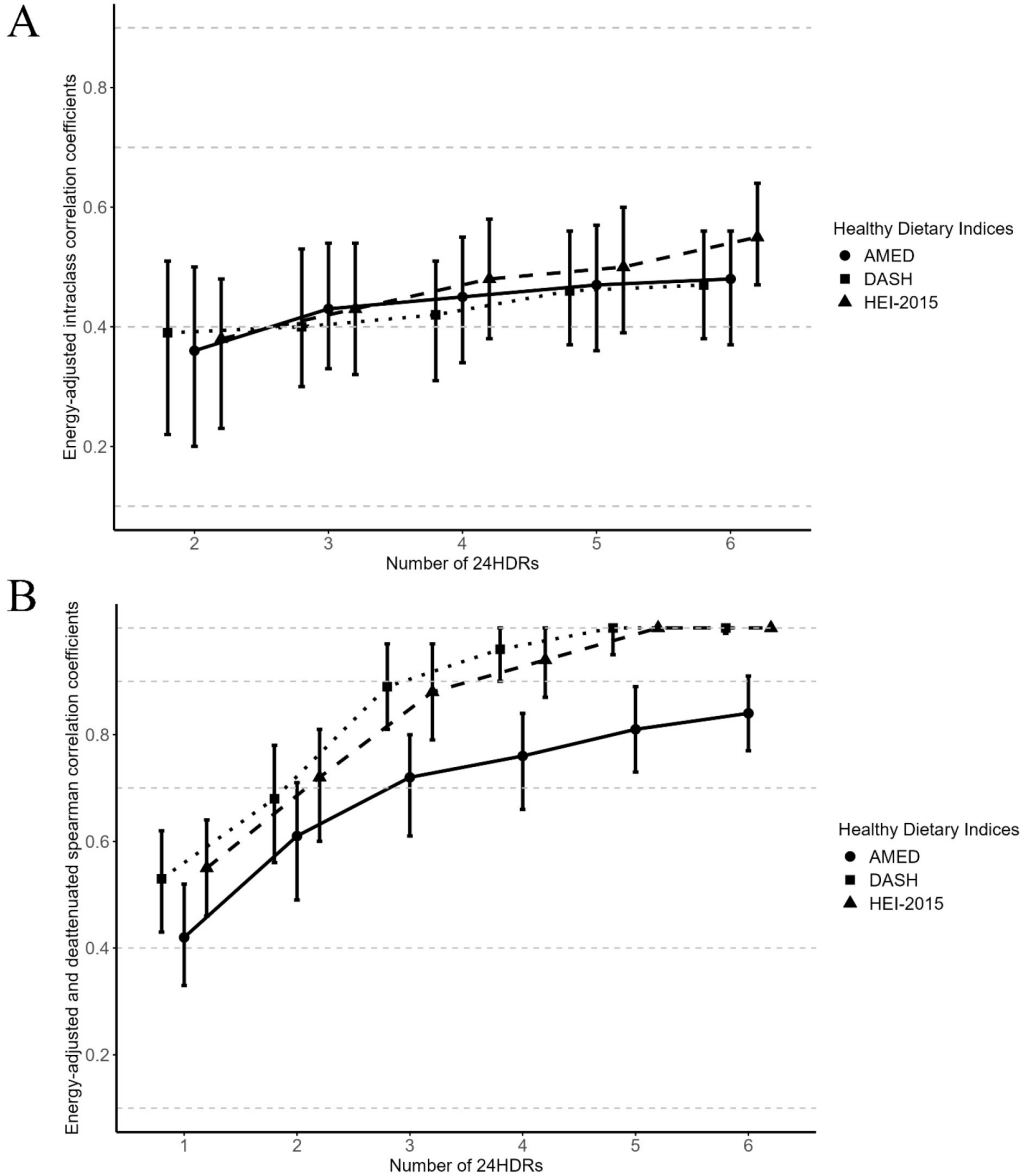
Bootstrap means and 95% CIs of energy-adjusted reproducibility coefficients (ICCs) and energy-adjusted validity coefficients (*r*_c_) for healthy dietary indices measured by different number of 24HDRs in the Anhui Lifestyle Validation Study (ALVS, 2021–2022). **(A)** The reproducibility of healthy dietary indices; **(B)** The validity of healthy dietary indices. The reproducibility of healthy dietary indices derived from 2, 3, 4, 5, and 6 24HDRs was assessed by energy-adjusted ICC. The validity was evaluated by comparing the estimates against those from the average of 12 24HDRs that consisted of four quarterly 3-day dietary recalls (i.e., two weekdays and one weekend day in every season) using energy-adjusted deattenuated spearman correlation coefficients. The coefficients and confidence interval limits above one were set to 1.00. The dashed lines represent the criteria for reproducibility and validity: “weak” (0.10–0.39), “moderate” (0.40–0.69), “strong” (0.70–0.89), and “very strong” (0.90–1.00). 24HDR, 24-hour dietary recall; ALVS, anhui lifestyle validation study; AMED, alternate Mediterranean diet; CIs, confidence intervals; DASH, dietary approaches to stop hypertension; HEI-2015, healthy eating index-2015; ICC, intraclass correlation coefficient; *r*_c_, deattenuated spearman correlation coefficients.

The reproducibility coefficients of healthy dietary indices were positively associated with the number of 24HDRs (Table 2 and Figure 1). We found that AMED, DASH, and HEI-2015 from three 24HDRs demonstrated “moderate” reproducibility, with ICCs of 0.43 (95% CI: 0.33–0.54), 0.40 (95% CI: 0.30–0.53), and 0.43 (95% CI: 0.32–0.54), respectively. All three healthy dietary indices estimated from four or more 24HDRs suggested a “moderate” reproducibility with ICCs ranging from 0.42 (DASH) to 0.55 (HEI-2015).

**Table 2:** Bootstrap means and 95% CIs of energy-adjusted reproducibility coefficients (ICC) and validity coefficients (*r*_*c*_) for healthy dietary indices measured by different number of 24HDRs in the Anhui Lifestyle Validation Study (ALVS, 2021–2022)^a^^b^.

Dietary indices	Energy-adjusted ICC (95% CI)	Energy-adjusted *r*_c_ (95% CI)^c^
AMED		
1 recall	—	0.42 (0.33, 0.52)
2 recalls	0.36 (0.20, 0.50)	0.61 (0.49, 0.71)
3 recalls	0.43 (0.33, 0.54)	0.72 (0.61, 0.80)
4 recalls	0.45 (0.34, 0.55)	0.76 (0.66, 0.84)
5 recalls	0.47 (0.36, 0.57)	0.81 (0.73, 0.89)
6 recalls	0.48 (0.37, 0.56)	0.84 (0.77, 0.91)
DASH		
1 recall	—	0.53 (0.43, 0.62)
2 recalls	0.39 (0.22, 0.51)	0.68 (0.56, 0.78)
3 recalls	0.40 (0.30, 0.53)	0.89 (0.81, 0.97)
4 recalls	0.42 (0.31, 0.51)	0.96 (0.90, 1.00)
5 recalls	0.46 (0.37, 0.56)	1.00 (0.95, 1.00)
6 recalls	0.47 (0.38, 0.56)	1.00 (0.99, 1.00)
HEI-2015		
1 recall	—	0.55 (0.46, 0.64)
2 recalls	0.38 (0.23, 0.48)	0.72 (0.60, 0.81)
3 recalls	0.43 (0.32, 0.54)	0.88 (0.79, 0.97)
4 recalls	0.48 (0.38, 0.58)	0.94 (0.87, 1.00)
5 recalls	0.50 (0.39, 0.60)	1.00 (1.00, 1.00)
6 recalls	0.55 (0.47, 0.64)	1.00 (1.00, 1.00)

24HDR, 24-hour dietary recall; ALVS, Anhui lifestyle validation study; AMED, alternate Mediterranean diet; CIs, confidence intervals; DASH, dietary approaches to stop hypertension; HEI-2015, healthy eating index-2015; ICC, intraclass correlation coefficient; *r*_*c*_, deattenuated spearman correlation coefficients.

^a^The reproducibility of healthy dietary indices derived from 2–6 non-consecutive 24HDRs was assessed using energy-adjusted ICC.

^b^nst those from the average of four quarterly three consecutive 24HDRs overall four seasons (two weekdays and one weekend day in each season) in 1 year using energy-adjusted deattenuated spearman correlation coefficients. Validity coefficients and confidence interval limits above one were set to 1.00.

^c^An *r*_*c*_ of one indicates extremely high validity of the dietary indices, reflecting complete agreement in individual ranking between estimates and not implying identical absolute values.

### 3.3 The validity of 1–6 non-consecutive 24HDRs

The validity coefficients increased with an escalating number of 24HDRs (Table 2 and Figure 1). After deattenuation for random measurement error, all three healthy dietary indices from one 24HDR showed “moderate” validity (*r*_c_: 0.42–0.55), “moderate” to “very strong” for 2–6 24HDRs (*r*_c_: 0.61–1.00). An *r*_c_ of one indicates extremely high validity of the dietary indices and reflects complete agreement in individual ranking between estimates, rather than identical absolute values.

The Bland-Altman plots illustrated a good agreement between three healthy dietary indices derived from three 24HDRs and those from the 12 24HDRs over 1-year, with the mean differences of 0.51 (LOA: −3.36 to 4.38) for AMED, −0.42 (LOA: −10.17 to 9.33) for DASH, and 0.07 (LOA: −7.64 to 7.79) for HEI-2015. For all three healthy dietary indices, measures form 3-day dietary recalls appeared to be overestimated among participants with lower adherence, but seemed to be underestimated among those with higher adherence (Supplementary Figure S2). Similar patterns were observed in 4-day (Supplementary Figure S3), 5-day (Supplementary Figure S4), and 6-day dietary recalls(Supplementary Figure S5).

### 3.4 The performance of three non-consecutive 24HDRs by individual characteristics

Since three non-consecutive 24HDR-based assessments of dietary indices showed reasonable reproducibility and validity in the analysis, we further evaluated their performance according to several potential influencing factors. Women seemed to have lower reproducibility of AMED compared to men (difference 59%, Table 3). Reproducibility of HEI-2015 was lower in participants BMI ≥24 kg/m^2^ than that in participants age <24 kg/m^2^ (differences 79%, Table 3). HEI-2015 showed a lower reproducibility coefficient in autumn/winter of administration than that in spring/summer of administration (difference 59%, Table 3). The validity coefficients of the three healthy dietary indices were similar between groups by age, sex, BMI, day of the week, and season of 24HDRs administration (difference within 20%, Table 4).

**Table 3:** Estimated bootstrap means and 95% CIs of energy-adjusted reproducibility coefficients (ICC) for healthy dietary indices measured by three non-consecutive 24HDRs according to individual characteristics in the Anhui lifestyle validation study (ALVS, 2021–2022)^a^.

Subgroup	AMED	*p*-value	DASH	*p*-value	HEI-2015	*p*-value
Age, years						
<45	0.35 (0.14, 0.54)	0.810	0.37 (0.17, 0.56)	0.126	0.38 (0.18, 0.55)	0.354
≥45	0.33 (0.16, 0.49)		0.32 (0.18, 0.45)		0.39 (0.24, 0.53)	
Sex						
Men	0.46 (0.32, 0.60)	0.146	0.34 (0.17, 0.48)	0.344	0.40 (0.21, 0.55)	0.934
Women	0.29 (0.11, 0.46)		0.44 (0.31, 0.61)		0.41 (0.25, 0.54)	
BMI, kg/m^2^						
<24	0.33 (0.16, 0.50)	0.782	0.32 (0.15, 0.47)	0.208	0.50 (0.37, 0.64)	0.036
≥24	0.30 (0.14, 0.47)		0.46 (0.31, 0.61)		0.28 (0.09, 0.44)	
Day of the week						
Weekday	0.36 (0.22, 0.47)	0.184	0.37 (0.26, 0.47)	0.390	0.32 (0.19, 0.44)	0.662
Weekend	0.23 (0.06, 0.36)		0.29 (0.13, 0.42)		0.28 (0.14, 0.40)	
Season						
Spring/summer	0.37 (0.24, 0.47)	0.664	0.49 (0.40, 0.58)	0.146	0.46 (0.36, 0.56)	0.028
Autumn/winter	0.40 (0.29, 0.50)		0.39 (0.27, 0.51)		0.29 (0.17, 0.42)	

24HDR, 24-hour dietary recall; ALVS, Anhui lifestyle validation study; AMED, alternate Mediterranean diet; BMI, body mass index; CIs, confidence intervals; DASH, dietary approaches to stop hypertension; HEI-2015, healthy eating index-2015; ICC, intraclass correlation coefficient.

^a^The reproducibility was evaluated by energy-adjusted ICC.

**Table 4:** Estimated bootstrap means and 95% CIs of energy-adjusted validity coefficients (*r*_c_) for healthy dietary indices measured by three non-consecutive 24HDRs according to individual characteristics in the Anhui lifestyle validation study (ALVS, 2021–2022)^a^.

Subgroup	AMED	*p*-value	DASH	*p*-value	HEI-2015	*p*-value
Age, years						
<45	0.69 (0.52, 0.85)	0.724	0.88 (0.75, 0.99)	0.916	0.84 (0.71, 0.94)	0.912
≥45	0.66 (0.50, 0.80)		0.86 (0.73, 0.96)		0.83 (0.71, 0.95)	
Sex						
Men	0.69 (0.53, 0.87)	0.564	0.93 (0.79, 1.00)	0.396	0.88 (0.73, 1.00)	0.532
Women	0.74 (0.61, 0.86)		0.86 (0.77, 0.99)		0.83 (0.72, 0.95)	
BMI, kg/m^2^						
<24	0.75 (0.59, 0.85)	0.804	0.95 (0.86, 1.00)	0.026	0.77 (0.62, 0.91)	0.126
≥24	0.71 (0.56, 0.83)		0.80 (0.65, 0.93)		0.89 (0.77, 0.98)	
Day of the week						
Weekday	0.59 (0.45, 0.72)	0.966	0.91 (0.82, 0.98)	0.522	1.00 (0.96, 1.00)	0.865
Weekend	0.57 (0.47, 0.71)		0.87 (0.77, 0.93)		1.00 (0.95, 1.00)	
Season						
Spring/summer	0.53 (0.40, 0.64)	0.188	0.85 (0.76, 0.94)	0.802	0.86 (0.78, 0.95)	0.008
Autumn/winter	0.63 (0.51, 0.74)		0.86 (0.77, 0.93)		0.98 (0.91, 1.00)	

24HDR, 24-hour dietary recall; ALVS, Anhui lifestyle validation study; AMED, alternate Mediterranean diet; BMI, body mass index; CIs, confidence intervals; DASH, dietary approaches to stop hypertension; HEI-2015, healthy eating index-2015; *r*_*c*_, deattenuated spearman correlation coefficients.

^a^The validity was evaluated by comparing the estimates against those from the average of 12 24HDRs that consisted of four quarterly 3-day dietary recalls (i.e., two weekdays and one weekend day in every season) using energy-adjusted deattenuated spearman correlation coefficients.

Bootstrap permutation tests showed that reproducibility of HEI-2015 differed by BMI (*p* = 0.036) and season (*p* = 0.028; Table 3), that validity of HEI-2015 differed by season (*p* = 0.008), and that validity of DASH differed by BMI (*p* = 0.026; Table 4).

## 4 Discussion

In this 1-year validation study of 432 Chinese free-living adults, we investigated the reproducibility and validity of the three healthy dietary indices (i.e., AMED, DASH, and HEI-2015) estimated from 24HDRs for the first time. Our findings suggest that three non-consecutive 24HDRs may have a reasonable performance in measuring the three dietary indices in epidemiological studies, while increasing the day of dietary recall above three appear not to substantially improve the reproducibility; however, validity continues to improve, peaking at four recall days. Additionally, higher BMI, female sex, and autumn/winter of administration were associated with low reproducibility of AMED and/or HEI-2015.

Although several studies (18–22) have validated the reproducibility and/or validity of different number of 24HDRs in dietary assessment, they have primarily focused on food groups or nutrient intake, with the recommended number of recalls differing across studies. For instance, St. George et al. (21). found that reliability estimates for assessing fruits and vegetables using three recalls ranged from 27 to 36% in African American youth. Another study (20) suggested that four recalls would be sufficient to provide reasonable reproducibility of reported food group (e.g., fruit and vegetables, milk group, and meat group) among peri-urban African adolescents. Only one study has specifically validated a healthy dietary index (the Chinese Healthy Eating Index) using three 24HDRs, showing good validity and reliability within the China Health and Nutrition Survey (39). However, to our knowledge, no studies have validated the reproducibility and validity of healthy dietary indices when considering multiple dietary recalls. In line with earlier work (18–21), our findings suggest that reliability and validity may improve as the number of recalls increases. Specifically, we found that three 24HDRs have an acceptable performance in measuring AMED, DASH, and HEI-2015.

Previous studies have evaluated the reproducibility and/or validity of healthy dietary indices derived from FFQs (12, 40–43). In the Men's Lifestyle Validation Study and Women's Lifestyle Validation Study in the United States, the reproducibility (ICC) for AMED and DASH scores derived from the FFQ ranged from 0.59 to 0.75, and the validity (deattenuated Spearman correlation coefficients) ranged from 0.56 to 0.77 (12). A study adapting HEI-2015 for the Brazilian population estimated a Cronbach's α of 0.65 (41). In Japan, a study assessing HEI-2015 reported reproducibility (ICC: 0.53–0.58) and validity (Spearman correlation: 0.43–0.57) (42).A study evaluating a short dietary screener in Singapore found reproducibility (ICC: 0.51–0.85) and validity (Spearman's rank-correlation coefficient: 0.47–0.61) for AHEI-2010, AMED, and DASH (40). Furthermore, an online Meal-based Diet History Questionnaire in Japan showed a median Spearman correlation of 0.43 for HEI-2015 (43). Our study demonstrated validity (*r*_c_: 0.72–0.89) and reproducibility (ICC: 0.40–0.44) from three 24HDRs, indicating reasonable performance in measuring AMED, DASH, and HEI-2015, supporting their use as a feasible alternative for dietary assessment in epidemiological research. Given the practicality and cost-effectiveness of FFQs in large epidemiological studies, they remain a widely used dietary assessment tool. However, the reliance on memory and the limited granularity of food items listed in FFQs can introduce recall bias and measurement error. In contrast, 24HDRs offer a more detailed account of day-to-day food consumption, capturing variations in portion sizes and cooking methods that FFQs may oversimplify or overlook. Nevertheless, in epidemiological studies, both FFQs and 24HDRs, if they show poor reproducibility and validity, can attenuate or obscure true diet-disease associations due to measurement error and reduced statistical power (35, 44, 45).

Results of the Bland-Altman plot suggested that participants with lower adherence to healthy diets tended to have their 3-day 24HDR-based scores overestimated, while those with higher adherence were more likely to be underestimated. One possible explanation is the “flattened slope” phenomenon, whereby individuals with extremely low or high intakes regress toward the mean over repeated measures (46). Especially in the context of assessing healthy dietary indices, social desirability bias may additionally encourage those with poorer diets to over-report healthier foods, while those who already follow healthier eating patterns might fail to capture occasional indulgences in their recalls (47). These dynamics reflect the complexity of dietary self-reporting and that future research could incorporate objective biomarkers (i.e., doubly labeled water or biomarkers of nutrient intake) into validation studies.

We observed differences in the reproducibility of three 24HDR-based assessments of HEI-2015 by BMI and season, with seasonal variation in validity, and differences in DASH validity by BMI. The lower reproducibility of HEI-2015 and lower validity of DASH among participants with BMI ≥24 kg/m^2^ may be attributed to higher dietary diversity among individuals with higher BMI and to reporting bias (48, 49). Seasonal fluctuations may affect dietary intake due to variations in the availability of fresh ingredients. Individuals may tend to prefer high-calorie foods to combat the cold in winter, whereas in summer, they may shift toward consuming more fruits and vegetables (50–52). These seasonal changes can impact the validity and reliability of HEI-2015 across different seasons, as HEI-2015 is based on healthy food groups such as fruits, vegetables, greens and beans, and whole grains, and places less emphasis on high-calorie foods like meat. Moreover, as the first two quarterly recalls were conducted in autumn and winter, participants had limited prior experience with the dietary recall procedures at these early time points, whereas by spring and summer they were more familiar with the interview process. Accordingly, the observed seasonal differences may not only reflect seasonal variation in dietary intake but may also be attributable to a learning effect, as participants were less familiar with the dietary recall procedures during the early stages of data collection.

The big strength of our study is the use of the average of 12 24HDRs that consisted of four quarterly 3-day dietary recalls (i.e., two weekdays and one weekend day in every season) over a year as the reference method in validity analysis, while considering the day of the week and seasonal variations in dietary assessment. Although non-consecutive recalls are generally considered preferable in settings with a single or limited number of assessment days to reduce within-person day-to-day variability, under the multi-time-point, multi-season repeated-measures design adopted in the present study, the short-term day-to-day correlation potentially introduced by consecutive recalls within each assessment period is expected to be effectively balanced at the annual level, thereby having a limited impact on the estimation of long-term habitual dietary intake. However, our study has some limitations. First, our study has not incorporated biomarkers or other objective measures of dietary intake to complement self-reported data, which may have resulted in the overestimation of validity coefficients, since random errors in both the reference method and the assessment tool (i.e., 24HDRs) might be positively correlated (53). Second, participants in the current study were community-dwelling adults from Central-Eastern China, which could restrict the applicability of the results to other populations given the different dietary culture in different populations. Therefore, our results need to be confirmed in future validation studies among diverse populations, ideally incorporating valid biomarkers or other objective measures (e.g., image-based food-recognition systems) into the study to correct measurement errors in the traditional assessment tools based on self-reporting (e.g., 24HDRs, dietary records, or FFQs), because these “objective” methods can reflect diet without being influenced by the same source of error (53).

In conclusion, our results support that three non-consecutive 24HDRs have moderate reproductivity and moderate to very strong validity in assessing AMED, DASH, and HEI-2015 in a Chinese population, while a single or two 24HDR(s) may not well capture the long-term adherence of these dietary indices. In addition, factors such as sex, BMI, and season may influence the reproducibility of these dietary indices. Future studies among multiple populations are warranted, ideally combined with objective measures of the diet to confirm the consistency and to adjust for measurement error if possible.

## Data Availability

Data described in the manuscript, code book, and analytic code will be made available upon request pending application.
