# Supplementary material for: Reproducibility and validity of healthy dietary indices derived from 24-hour dietary recalls
Source: Front Nutr. 2026 Apr 2;13:1730154. doi: 10.3389/fnut.2026.1730154 (PMC13082945; doi:10.3389/fnut.2026.1730154)
Supplement: Supplementary file 1 [file Table_1.docx]

Supplementary Material

**Reproducibility and validity of healthy dietary indices derived from 24-hour dietary recalls**

**Supplementary method 1** Assessment of healthy dietary indices

**Supplementary method 2** Calculation of the deattenuated spearman correlation coefficients (*r_c_*)

**Table S1** Components, scoring methods, and ranges of AMED

**Table S2** Components, scoring methods, and ranges of DASH

**Table S3** Components, scoring methods, and ranges of HEI-2015

**Table S4** Median (interquartile range) scores for healthy dietary indices derived from different number of 24HDRs in the Anhui Lifestyle Validation Study (ALVS, 2021–2022)

**Fig. S1** Flowchart of participant selection in the Anhui Lifestyle Validation Study (ALVS, 2021–2022)

**Fig. S2** Bland-Altman plot illustrating the agreement between 3 randomly non-consecutive 24HDRs and 4 quarterly 3 consecutive 24HDRs overall 4 seasons (2 weekdays and 1 weekend day in each season) in 1 year when estimating healthy dietary indices in the Anhui Lifestyle Validation Study (ALVS, 2021–2022)

**Fig. S3** Bland-Altman plot illustrating the agreement between 4 randomly non-consecutive 24HDRs and 4 quarterly 3 consecutive 24HDRs overall 4 seasons (2 weekdays and 1 weekend day in each season) in 1 year when estimating healthy dietary indices in the Anhui Lifestyle Validation Study (ALVS, 2021–2022)

**Fig. S4** Bland-Altman plot illustrating the agreement between 5 randomly non-consecutive 24HDRs and 4 quarterly 3 consecutive 24HDRs overall 4 seasons (2 weekdays and 1 weekend day in each season) in 1 year when estimating healthy dietary indices in the Anhui Lifestyle Validation Study (ALVS, 2021–2022)

**Fig. S5** Bland-Altman plot illustrating the agreement between 6 randomly non-consecutive 24HDRs and 4 quarterly 3 consecutive 24HDRs overall 4 seasons (2 weekdays and 1 weekend day in each season) in 1 year when estimating healthy dietary indices in the Anhui Lifestyle Validation Study (ALVS, 2021–2022)

**Supplementary method 1. Assessment of healthy dietary indices**

The AMED score is based on 9 components, with 1 point given for consumption above the sex-specific median for 7 components (vegetables (excluding potatoes), whole grains, fruits, legumes, nuts, fish, ratio of monounsaturated to saturated fat). Conversely, 1 point is awarded for consumption below the sex-specific median for red and processed meat. Participants receive 0 points if their consumption does not meet these criteria. For alcohol consumption, 1 point is assigned to women (5–15 g/day) and men (10–25 g/day), with 0 points given for consumption outside these ranges.(1) The AMED score ranges from 0 to 9 points (**Table S1**).

The DASH score includes 8 components, assigning points from 1 (lowest intake, quintile 1) to 5 (highest intake, quintile 5) for sex-specific consumption levels of fruits, vegetables (excluding potatoes), nuts and legumes, whole grains, and low-fat dairy products. Reverse scoring is applied to red and processed meat, sodium, and sugar-sweetened beverages.(1)

Due to salt data is unavailable in the 24HDRs, sodium intake was estimated based on the sodium content of foods.(2) The DASH score ranges from 8 to 40 points (**Table S2**).

The HEI-2015 contains 9 adequacy components and 4 moderation components,(3) consistent with the HEI-2020.(4) Added sugars was not included as a component of the HEI-2015 in our study, due to the lack of relevant data. The 3 adequacy components (whole grains, dairy, and fatty acids) were scored 0 to 10 points. The remaining 6 adequacy components (total fruits, whole fruits, total vegetables, greens and beans, total protein foods, and seafood and plant proteins) were scored 0 to 5 points. The 3 moderation components (refined grains, sodium, and saturated sats), which are recommended to be limited, are scored 0 to 10 points each. The HEI-2015 ranges from 0 to 90 points (**Table S3**). Higher scores represent greater adherence to healthy dietary indices.

**Supplementary method 2.** **Calculation of the deattenuated Spearman correlation coefficients (*r_c_*)**

The *r_c_* for a single randomly selected 24HDR compared to the 12 24HDRs was calculated using the following formula:

*r_c_* = *r_0_*$\sqrt{1+/k}$

where *r_c_* is the deattenuated Spearman correlation coefficient, *r_0_*​ is the observed Spearman correlation coefficient, is the ratio of within-subject to between-subject variance, and *k* is the number of repeated measurements (12 days in this study).

For comparisons involving 2 to 6 randomly selected 24HDRs against the 12 24HDRs, the *r_c_* was determined using this formula:

*r_c_* = *r_0_*$\sqrt{(1+{}_{x}/k_{x})(1+{}_{y}/k_{y})}$

where *r_c_* is the deattenuated Spearman correlation coefficient, *r_0_*_​_ is the observed Spearman correlation coefficient, ${}_{x}$ and ${}_{y}$are the ratios of within-subject to between-subject variance for the two variables, and$k_{x}$​ and $k_{y}$​ are the number of repeated measurements for the two variables (with the reference variable measured over 12 days in this study).

**Table S1** Components, scoring methods, and ranges of AMED

| **Component** |  | **Criteria for minimum score of 0** |  | **Criteria for maximum score of 1** |  | **Score Range** |
| --- | --- | --- | --- | --- | --- | --- |
| Vegetables (excluding potatoes), g/d |  | Less than median |  | Median or greater |  | 0 or 1 |
| Fruits, g/d |  | Less than median |  | Median or greater |  | 0 or 1 |
| Whole grains, g/d |  | Less than median |  | Median or greater |  | 0 or 1 |
| Nuts, g/d |  | Less than median |  | Median or greater |  | 0 or 1 |
| Legumes, g/d |  | Less than median |  | Median or greater |  | 0 or 1 |
| Red and processed meat, g/d |  | Median or greater |  | Less than median |  | 1 or 0 |
| Fish, g/d |  | Less than median |  | Median or greater |  | 0 or 1 |
| Ratio of monounsaturated to saturated fat |  | Less than median |  | Median or greater |  | 0 or 1 |
| Alcohol, g/d |  |  |  |  |  |  |
| Women |  | <5 or >15 |  | 5–15 |  | 1 or 0 |
| Men |  | <10 or >25 |  | 10–25 |  | 1 or 0 |
| Total |  |  |  |  |  | 0–9 |

AMED, Alternate Mediterranean Diet.

Table S2 Components, scoring methods, and ranges of DASH

| **Component** |  | **Criteria for minimum score of 1** |  | **Criteria for maximum score of 5** |  | **Score Range** |
| --- | --- | --- | --- | --- | --- | --- |
| Vegetables (excluding potatoes and legumes), g/d |  | Lowest quintile |  | Highest quintile |  | 1–5 |
| Fruit, g/d |  | Lowest quintile |  | Highest quintile |  | 1–5 |
| Nut and legumes, g/d |  | Lowest quintile |  | Highest quintile |  | 1–5 |
| Whole grains, g/d |  | Lowest quintile |  | Highest quintile |  | 1–5 |
| Low fat dairy, g/d |  | Lowest quintile |  | Highest quintile |  | 1–5 |
| Red and processed meat, g/d |  | Highest quintile |  | Lowest quintile |  | 1–5 |
| Sugar-sweetened beverages, g/d |  | Highest quintile |  | Lowest quintile |  | 1–5 |
| Sodium, mg/d |  | Highest quintile |  | Lowest quintile |  | 1–5 |
| Total |  |  |  |  |  | 8–40 |

DASH, Dietary Approaches to Stop Hypertension.

**Table S3** Components, scoring methods, and ranges of HEI-2015^a^

| **Component** |  | **Standard for maximum score** |  | **Standard for minimum score of 0** |  | **Score Range** |
| --- | --- | --- | --- | --- | --- | --- |
| **Adequacy components** | | | | | | |
| Total Fruits |  | ≥0.8 cup equivalents per 1000 kcal |  | No Fruits |  | 0–5 |
| Whole Fruits |  | ≥0.4 cup equivalents per 1000 kcal |  | No Whole Fruits |  | 0–5 |
| Total Vegetables |  | ≥1.1 cup equivalents per 1000 kcal |  | No Vegetables |  | 0–5 |
| Greens and Beans |  | ≥0.2 cup equivalents per 1000 kcal |  | No Greens and Beans |  | 0–5 |
| Whole Grains |  | ≥1.5 oz equivalents per 1000 kcal |  | No Whole Grains |  | 0–10 |
| Dairy |  | ≥1.3 cup equivalents per 1000 kcal |  | No Dairy |  | 0–10 |
| Total Protein Foods |  | ≥2.5 oz equivalents per 1000 kcal |  | No Protein Foods |  | 0–5 |
| Seafood and Plant Proteins |  | ≥0.8 oz equivalents per 1000 kcal |  | No Seafood or Plant Proteins |  | 0–5 |
| Fatty Acids |  | (PUFAs + MUFAs)/SFAs ≥2.5 |  | (PUFAs + MUFAs)/SFAs ≤1.2 |  | 0–10 |
| **Moderation components** | | | | | | |
| Refined Grains |  | ≤1.8 oz equivalents per 1000 kcal |  | ≥4.3 oz equivalents per 1000 kcal |  | 0–10 |
| Sodium |  | ≤1.1 gram per 1000 kcal |  | ≥2.0 grams per 1000 kcal |  | 0–10 |
| Added Sugars |  | ≤6.5% of energy |  | ≥26% of energy |  | NA |
| Saturated Fats |  | ≤8% of energy |  | ≥16% of energy |  | 0–10 |
| **Total** |  |  |  |  |  | 0–90 |

HEI-2015, Healthy Eating Index-2015; MUFA, Monounsaturated Fatty Acid; PUFA, Polyunsaturated Fatty Acid; SFA, Saturated Fatty Acid.

^a^All standards in HEI-2015 represent amounts per 1000 kcal (sometimes shown as percentage of energy) except for fatty acids.

**Table S4** Median (interquartile range) scores for healthy dietary indices derived from different number of 24HDRs in the Anhui Lifestyle Validation Study (ALVS, 2021–2022)^ab^

| Dietary recalls |  | AMED | |  | DASH | |  | HEI-2015 | |
| --- | --- | --- | --- | --- | --- | --- | --- | --- | --- |
|  |  | Score | Diff (%) |  | Score | Diff (%) |  | Score | Diff (%) |
| 1 recall |  | 6.0 (4.0–8.0) | 22.4 |  | 24.2 (20.0–28.2) | 0.8 |  | 34.7 (30.8–39.6) | 5.2 |
| 2 recalls |  | 6.2 (4.3–6.4) | 26.5 |  | 22.7 (18.8–28.3) | 5.4 |  | 35.9 (32.7–39.8) | 1.7 |
| 3 recalls |  | 6.2 (4.3–6.5) | 26.5 |  | 24.3 (18.5–28.5) | 1.3 |  | 36.7 (33.6–41.2) | 0.5 |
| 4 recalls |  | 5.5 (4.5–6.6) | 12.2 |  | 23.9 (20.0–28.2) | 0.4 |  | 36.2 (33.3–40.5) | 0.8 |
| 5 recalls |  | 6.2 (4.4–6.6) | 26.5 |  | 23.9 (19.1–28.3) | 0.4 |  | 44.3 (39.8–48.5) | 21.4 |
| 6 recalls |  | 6.2 (4.4–6.6) | 26.5 |  | 24.3 (18.3–28.3) | 1.3 |  | 36.3 (33.7–40.5) | 0.5 |
| 12 recalls |  | 4.9 (3.1–6.9) | - |  | 24.0 (20.0–28.1) | - |  | 36.5 (33.9–40.0) | - |

24HDR, 24-Hour Dietary Recall; ALVS, Anhui Lifestyle Validation Study; AMED, Alternate Mediterranean Diet; DASH, Dietary Approaches to Stop Hypertension; HEI-2015, Healthy Eating Index-2015.

^a^Healthy dietary indices were energy adjusted by residual method.

^b^The differences were calculated as the median scores for healthy dietary indices obtained from different number of dietary recalls minus the reference method (12 24HDRs). Diff (%) represents the absolute value of difference as a percentage of the median scores from 12 recalls.

Anhui Lifestyle Validation Study (ALVS), Huoshan, China

*n* = 754

*n* = 459

Individuals were excluded if they did not participate in the 12 24HDRs (*n* = 295)

Individuals were excluded if they had implausible energy intake (*n* = 27)

*n* = 432

Available for the analysis

**Fig. S1** Flowchart of participant selection in the Anhui Lifestyle Validation Study (ALVS, 2021–2022). 24HDR, 24-Hour Dietary Recall; ALVS, Anhui Lifestyle Validation Study.


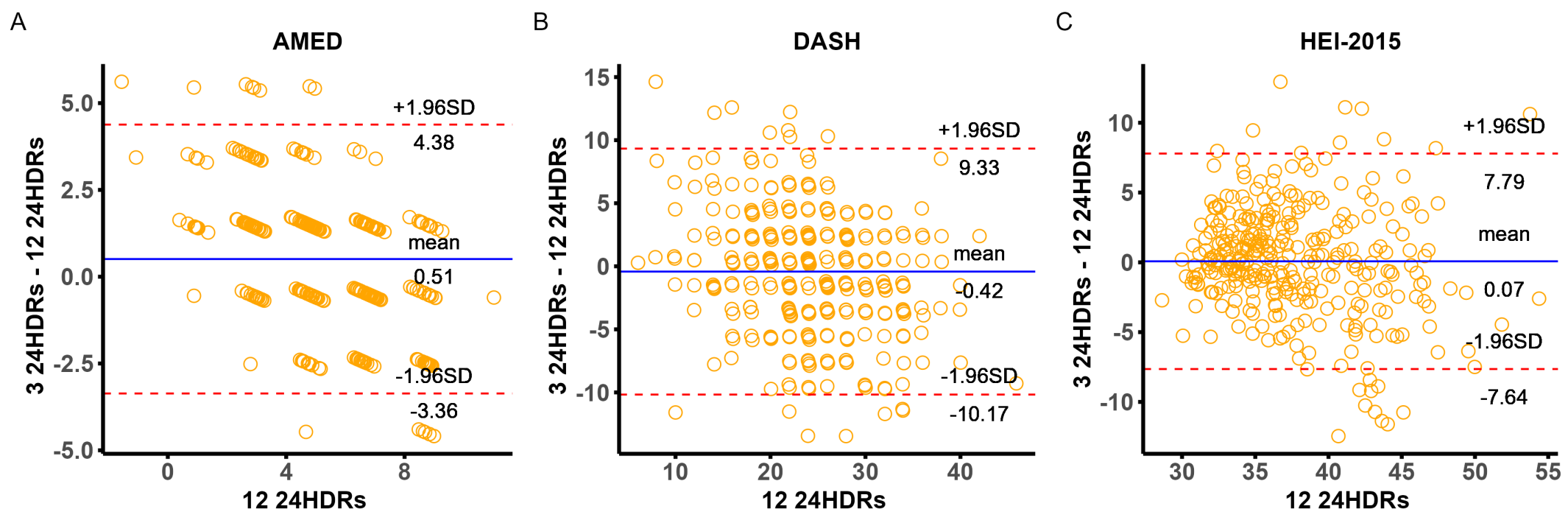


**Fig. S2** Bland-Altman plot illustrating the agreement between 3 randomly non-consecutive 24HDRs and 4 quarterly 3 consecutive 24HDRs overall 4 seasons (2 weekdays and 1 weekend day in each season) in 1 year when estimating healthy dietary indices in the Anhui Lifestyle Validation Study (ALVS, 2021–2022). The mean difference and the 95% limits of agreement (LOA) are depicted by 3 horizontal lines. (A) AMED; (B) DASH; (C) HEI-2015. 24HDR, 24-Hour Dietary Recall; ALVS, Anhui Lifestyle Validation Study; AMED, Alternate Mediterranean Diet; DASH, Dietary Approaches to Stop Hypertension; HEI-2015, Healthy Eating Index-2015; LOA, Limits of Agreement; SD, Standard Deviation.

**
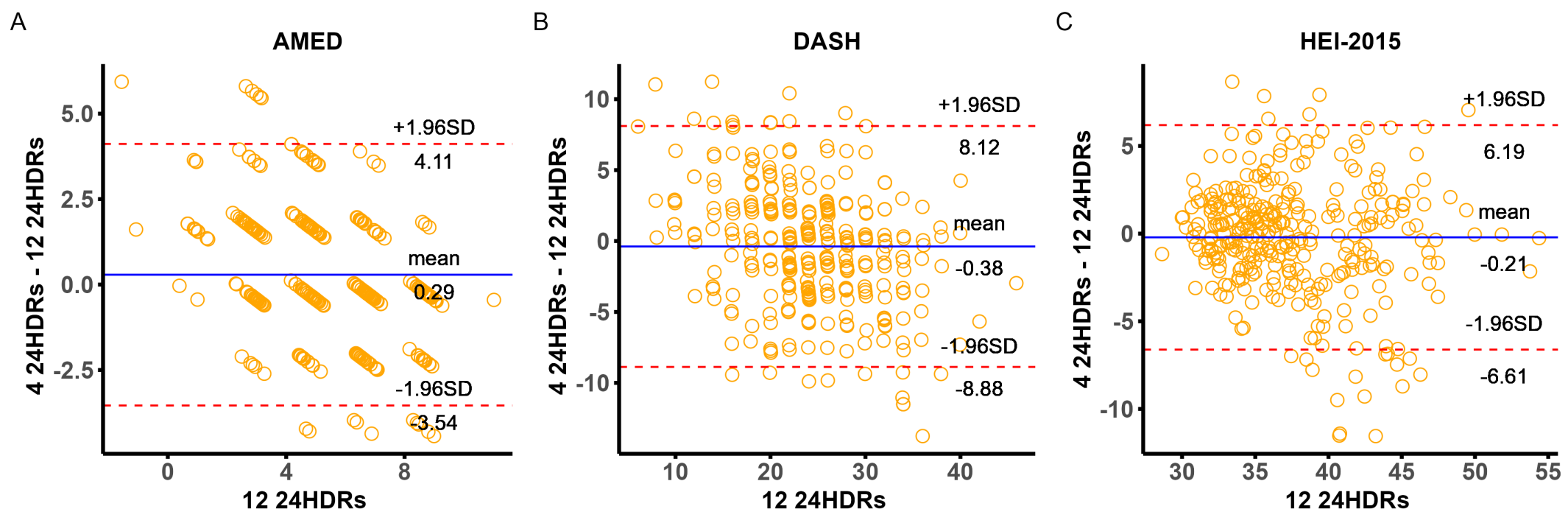
Fig. S3** Bland-Altman plot illustrating the agreement between 4 randomly non-consecutive 24HDRs and 4 quarterly 3 consecutive 24HDRs overall 4 seasons (2 weekdays and 1 weekend day in each season) in 1 year when estimating healthy dietary indices in the Anhui Lifestyle Validation Study (ALVS, 2021–2022). The mean difference and the 95% limits of agreement (LOA) are depicted by 3 horizontal lines. (A) AMED; (B) DASH; (C) HEI-2015. 24HDR, 24-Hour Dietary Recall; ALVS, Anhui Lifestyle Validation Study; AMED, Alternate Mediterranean Diet; DASH, Dietary Approaches to Stop Hypertension; HEI-2015, Healthy Eating Index-2015; LOA, Limits of Agreement; SD, Standard Deviation.


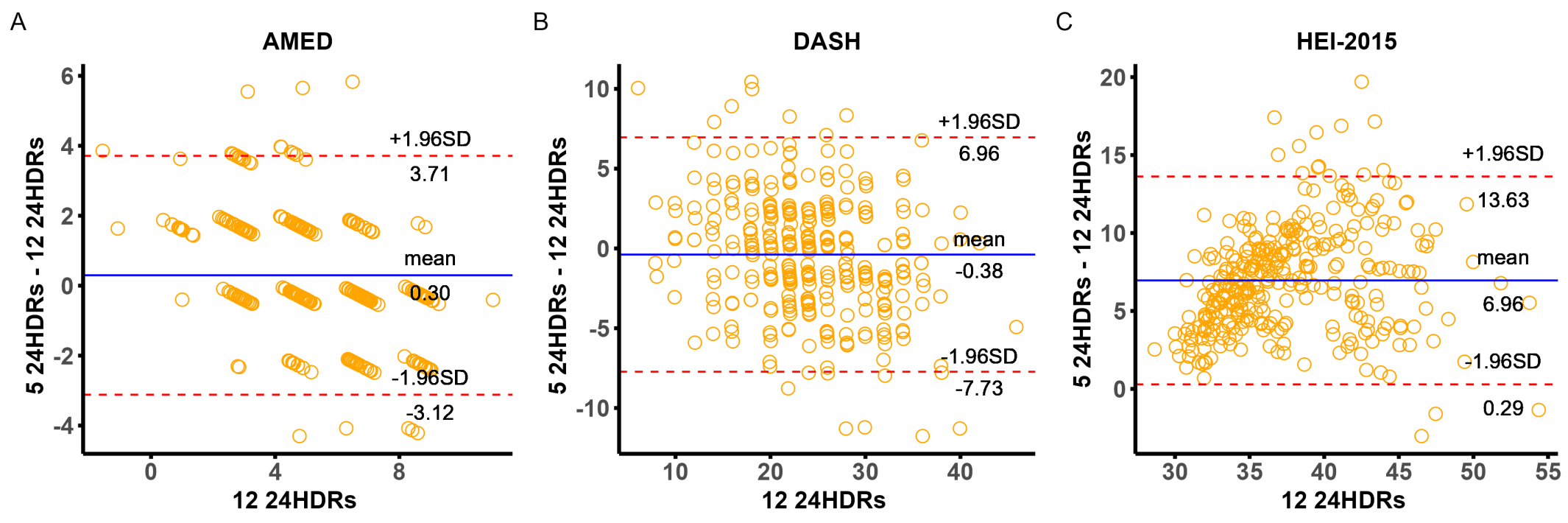


**Fig. S4** Bland-Altman plot illustrating the agreement between 5 randomly non-consecutive 24HDRs and 4 quarterly 3 consecutive 24HDRs overall 4 seasons (2 weekdays and 1 weekend day in each season) in 1 year when estimating healthy dietary indices in the Anhui Lifestyle Validation Study (ALVS, 2021–2022). The mean difference and the 95% limits of agreement (LOA) are depicted by 3 horizontal lines. (A) AMED; (B) DASH; (C) HEI-2015. 24HDR, 24-Hour Dietary Recall; ALVS, Anhui Lifestyle Validation Study; AMED, Alternate Mediterranean Diet; DASH, Dietary Approaches to Stop Hypertension; HEI-2015, Healthy Eating Index-2015; LOA, Limits of Agreement; SD, Standard Deviation.


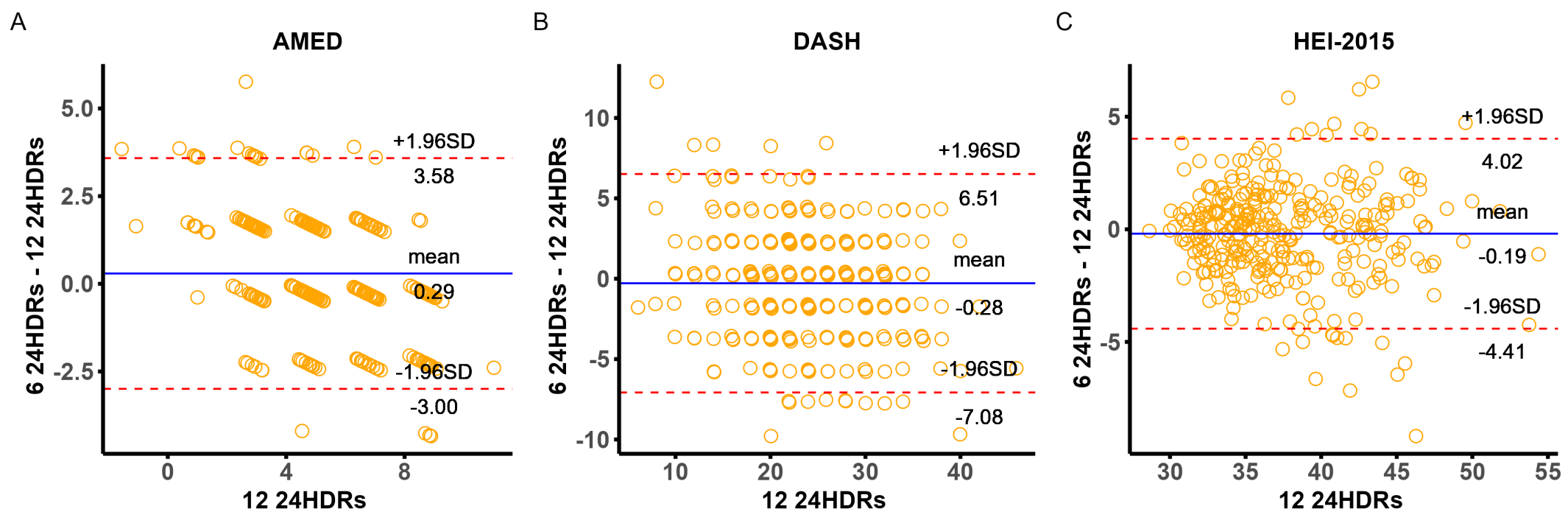


**Fig. S5** Bland-Altman plot illustrating the agreement between 6 randomly non-consecutive 24HDRs and 4 quarterly 3 consecutive 24HDRs overall 4 seasons (2 weekdays and 1 weekend day in each season) in 1 year when estimating healthy dietary indices in the Anhui Lifestyle Validation Study (ALVS, 2021–2022). The mean difference and the 95% limits of agreement (LOA) are depicted by 3 horizontal lines. (A) AMED; (B) DASH; (C) HEI-2015. 24HDR, 24-Hour Dietary Recall; ALVS, Anhui Lifestyle Validation Study; AMED, Alternate Mediterranean Diet; DASH, Dietary Approaches to Stop Hypertension; HEI-2015, Healthy Eating Index-2015; LOA, Limits of Agreement; SD, Standard Deviation.

**Reference**

1. Ma Y, Yang W, Simon TG, Smith-Warner SA, Fung TT, Sui J, Chong D, VoPham T, Meyerhardt JA, Wen D, Giovannucci EL, Chan AT, Zhang X. Dietary Patterns and Risk of Hepatocellular Carcinoma Among U.S. Men and Women. Hepatology. 2019 Aug;70(2):577-586. Epub 2018/12/07. doi:10.1002/hep.30362. Cited in: Pubmed; PMID 30506561.

2. Xiao ML, Lin JS, Li YH, Liu M, Deng YY, Wang CY, Chen YM. Adherence to the Dietary Approaches to Stop Hypertension (DASH) diet is associated with lower presence of non-alcoholic fatty liver disease in middle-aged and elderly adults. Public Health Nutr. 2020 Mar;23(4):674-682. Epub 2019/10/01. doi:10.1017/S1368980019002568. Cited in: Pubmed; PMID 31566148.

3. Krebs-Smith SM, Pannucci TE, Subar AF, Kirkpatrick SI, Lerman JL, Tooze JA, Wilson MM, Reedy J. Update of the Healthy Eating Index: HEI-2015. J Acad Nutr Diet. 2018 Sep;118(9):1591-1602. Epub 2018/08/28. doi:10.1016/j.jand.2018.05.021. Cited in: Pubmed; PMID 30146071.

4. Shams-White MM, Pannucci TE, Lerman JL, Herrick KA, Zimmer M, Meyers Mathieu K, Stoody EE, Reedy J. Healthy Eating Index-2020: Review and Update Process to Reflect the Dietary Guidelines for Americans,2020-2025. J Acad Nutr Diet. 2023 Sep;123(9):1280-1288. Epub 2023/05/19. doi:10.1016/j.jand.2023.05.015. Cited in: Pubmed; PMID 37201748.
